# Analysis of drug treatment outcome in clarithromycin-resistant *Mycobacterium avium* complex lung disease

**DOI:** 10.1186/s12879-016-1384-7

**Published:** 2016-01-27

**Authors:** Tsukasa Kadota, Hirotoshi Matsui, Takashi Hirose, Junko Suzuki, Minako Saito, Tomohiro Akaba, Kouichi Kobayashi, Shunsuke Akashi, Masahiro Kawashima, Atsuhisa Tamura, Hideaki Nagai, Shinobu Akagawa, Nobuyuki Kobayashi, Ken Ohta

**Affiliations:** 1 Center for Pulmonary Diseases, National Hospital Organization Tokyo National Hospital, 3-1-1, Takeoka, Kiyose-shi, Tokyo 204-8585 Japan; 2 Division of Respiratory Disease, Department of Internal Medicine, The Jikei University School of Medicine, 3-25-8, Nishi-Shimbashi, Minato-ku, Tokyo 105-8461 Japan; 3 Asthma and Allergy Center, National Hospital Organization Tokyo National Hospital, 3-1-1, Takeoka, Kiyose-shi, Tokyo 204-8585 Japan

**Keywords:** Mycobacterium avium complex, Drug resistance, Macrolides, Drug therapy

## Abstract

**Background:**

Although the isolation of clarithromycin (CAM)-resistant Mycobacterium avium complex (MAC) indicates a poor treatment outcome and increased mortality, there have been only a few reports on drug treatment for CAM-resistant MAC lung disease. We aimed to reveal the effectiveness of the continuation of a macrolide and the use of a multidrug regimen in the treatment of CAM-resistant MAC lung disease.

**Methods:**

Among patients with MAC pulmonary disease as defined by the 2007 criteria of the American Thoracic Society and the Infectious Diseases Society of America statement, those with CAM-resistant MAC (minimum inhibitory concentration ≥32 μg/ml) isolated, newly diagnosed and treated from January 2009 to June 2013 were analysed in this study. Effectiveness was measured based on culture conversion rate and improvement of radiological findings.

**Results:**

Thirty-three HIV-negative patients were analysed in this study. Twenty-six were treated with a regimen containing CAM or azithromycin (AZM), and 21 patients were treated with three or more drugs except macrolide. The median duration to be evaluated was 10.4 months after beginning the treatment regimen. Sputum conversion (including cases of inability to expectorate sputum) was achieved in 12 (36 %) patients. Radiological effectiveness improved in 4 (12 %) patients, was unchanged in 11 (33 %) patients and worsened in 18 (55 %) patients. In the multivariate analysis, CRP <1.0 mg/dl (*p* = 0.017, odds ratio 12, 95 % confidence interval (CI) 1.6–95) was found to be the only significant risk factor for radiological non-deterioration, and no significant risk factors for microbiological improvement were found.

**Conclusions:**

Our results suggested that continuation of macrolides or the addition of a new quinolone or injectable aminoglycoside to therapy with rifampicin and ethambutol would not improve clinical outcome after the emergence of CAM-resistant MAC. However, further prospective study is required to evaluate the precise clinical efficacy and effectiveness of these drugs.

## Background

*Mycobacterium avium* complex (MAC) is increasingly important as a causative agent of pulmonary disease [1–3]. Drug therapy for MAC lung disease is long, costly and often associated with drug-related toxicity. Current treatment recommendations for MAC lung disease include a macrolide such as clarithromycin (CAM) or azithromycin (AZM), ethambutol (EB) and a rifamycin such as rifampicin (RFP) or rifabutin (RBT) [1]. Among these drugs, macrolides are the only antimicrobial agents for which there has been demonstrated correlation between in vitro susceptibility and clinical response for MAC lung disease [4, 5]. Because the isolation of CAM-resistant MAC indicates a poor treatment outcome and increased mortality, preventing the risk factors that may have contributed to the development of MAC with acquired drug resistance is very important. E.g. macrolide monotherapy, macrolide plus only a quinolone and unnecessary long-term macrolide combination regimen [4, 6]. However, now that long-term macrolide monotherapy has been found to be beneficial for patients with pulmonary diseases such as cystic fibrosis [7], panbronchiolitis [8], chronic obstructive pulmonary disease (COPD) [9, 10], and non-cystic fibrosis bronchiectasis [11, 12], the importance of the treatment of CAM-resistant MAC lung disease would be increasing.

The treatment of CAM-resistant MAC lung disease is either by drug therapy only or by surgery and drug therapy. Although a previous study showed that the use of both aminoglycosides and surgical resection of involved lung tissue resulted in relatively good clinical effectiveness, patients who can be candidates for surgery are limited [6]. As for drug therapy, discontinuation of the macrolide and the addition of a parenteral agent (aminoglycoside, e.g., kanamycin [KM]) and/or a new quinolone might be considered. However, there have been only a few reports on drug treatment regimens for patients with CAM-resistant MAC lung disease. To our knowledge, no published study has focused on the effectiveness of the continuation of a macrolide and the use of a multidrug regimen including a parenteral aminoglycoside and a new quinolone. Therefore, we aimed to reveal the effectiveness of the continuation of a macrolide and the use of a multidrug regimen in the treatment of CAM-resistant MAC lung disease.

## Methods

### Study population

MAC pulmonary disease was defined by the 2007 criteria of the American Thoracic Society and the Infectious Diseases Society of America (ATS/IDSA) statement [1]. Subsequently, patients with newly diagnosed CAM-resistant MAC (minimum inhibitory concentration [MIC] ≥32 μg/ml) isolated from sputum and treated at Tokyo National Hospital from January 2009 to June 2013 were analysed in this study. Patients treated for less than 5 months with a single regimen were excluded from this study. We retrospectively reviewed medical records, chest X-ray and computed tomography (CT) scans, and microbiological examinations.

### Microbiological examination

Microbiological examination was performed with standard methods [13]. The sputum was directly examined with Ziehl-Neelsen staining. Specimens submitted for culture were digested and decontaminated by sodium hydroxide, and the samples were inoculated onto slants of 2 % Ogawa egg medium (Japan BCG, Tokyo, Japan) or Mycobacteria Growth Indicator Tube and then identified by growth characteristics, conventional biochemical tests and molecular method by transcription-reverse transcription concerted reaction assay (Tosoh Bioscience, Tokyo, Japan).

Antimicrobial susceptibility testing was performed according to the broth microdilution methods as described by the Clinical Laboratory Standards Institute [14]. The antimicrobial agents tested included CAM, RFP, EB, levofloxacin (LVFX), KM, streptomycin (SM) and amikacin (AMK). The drug concentration ranges for tested drugs were as follows: CAM, 0.5 to 32 μg/ml; RFP, 0.125 to 16 μg/ml; EB, 2 to 128 μg/ml; LVFX, 0.25 to 16 μg/ml; SM, 0.5 to 16 μg/ml; and AMK, 2 to 32 μg/ml. Isolates were considered resistant if the MICs of RFP, EB, LVFX, KM, SM and AMK were 8 μg/ml or greater [15, 16].

### Radiological examination

Radiographic abnormalities were classified as showing either nodular bronchiectatic disease or fibrocavitary disease on the basis of chest radiography and high-resolution CT findings at the initial diagnosis of CAM-resistant MAC. Lesion extent was also evaluated based on chest radiographs and the total extent was defined as follows: 1 (within one third of the unilateral lung field), 2 (within the unilateral lung field), and 3 (over the entire unilateral lung field) [17, 18].

### Antibiotic treatment

Patients with CAM-resistant MAC lung disease received combination oral and parental antibiotic therapy. For most patients, the regimen included CAM at 600–800 mg/day, EB at 15 mg/kg/day, RFP at 450 mg/day (if body weight <50 kg) or 600 mg/day, and KM 15 mg/kg intramuscularly given 2 or 3 times a week for the initial 5–6 months of therapy and subsequent daily new quinolone. As the new quinolone, LVFX 500 mg/day, moxifloxacin (MFLX) 400 mg/day and sitafloxacin (STFX) 100 mg/day were used. The regimen in this study was defined as the first regimen continued for more than 5 months after the confirmation of CAM-resistant MAC.

### Assessment of clinical effectiveness

Clinical effectiveness was measured based on the sputum conversion rate and improvement of radiological findings during 12 months of treatment. Sputum conversion was defined as two consecutive negative cultures. If the patient could not expectorate sputum, the sputum was considered to have converted to negative [5, 17, 19]. Radiological effectiveness was divided into 3 categories by three of the authors (T. K., H. M. and T. H.) who reviewed the radiographs. The reviewers were blined to the treatment regimen and sputum conversion. ‘Improved’ was defined as a decrease in the abnormal shadows due to pulmonary MAC; ‘unchanging’ was defined as abnormal shadows that were almost the same as those before the initiation of CAM-resistant MAC lung disease treatment; and ‘worsening’ was defined as an increase in abnormal shadows compared with those of the lesions before the treatment. Differences in classification between the authors were resolved by majority vote.

### Statistical analysis

All results are presented as means ± standard deviation (SD) and as numbers (percentages). The Fisher’s exact test was used for the comparison of the variables. For the multivariable models, the variables with *p* values of <0.20 in the univariate models and the variables of drug treatment were entered into the logistic regression analysis. We then evaluated using forced entry logistic regression analysis to estimate odds ratios (OR). A two-sided *p* value of <0.05 was considered to indicate a statistically significant difference for all analyses. All analyses were performed with SPSS statistical software (SPSS version 20, Chicago, IL, USA).

### Ethics statement

Individual written informed consent for this research were not required since the present study was a retrospective observational study by reviewing medical records. The Ethics Committee of Tokyo National Hospital approved this study as it is (IRB No.130060).

## Results

### Patient characteristics and antibiotic treatment

From January 2009 to June 2013, 38 patients were newly diagnosed with CAM-resistant MAC isolated from sputum and treated at Tokyo National Hospital. Of these, we excluded five cases from the present study because of treatment less than 5 months with a single regimen in three cases, loss of follow-up in one case and death within 5 months after the isolation of CAM-resistant MAC in one case. Therefore, 33 patients with CAM-resistant MAC lung disease were analysed in this study. Patients’ characteristics are shown in Table 1. Of these 33 patients, 31 (94 %) were women (mean age, 67 years), and 30 (91 %) had never smoked. Also, there are no patients with HIV infection, extrapulmonary MAC disease, infection from other nontuberculous mycobacteria and malignancy. We did not performed bronchoscopy in patients with clinical or radiologic worsening of disease. Drug susceptibility of the MAC isolates is shown in Table 2. Of the oral antibiotics, RFP and LVFX displayed in vitro activity against most isolates. Of the parenteral antibiotics, SM demonstrated better in vitro activity than KM and AMK.

**Table 1:** Characteristics of the 33 patients

Variables	Total (*n* = 33)
Age at onset of MAC, yrs ± SD	57 ± 12
Age (when found to be resistant), yrs ± SD	67 ± 9
Female, n (%)	31 (93.9)
Body mass index, kg/m^2^ ± SD	17.2 ± 5.0
Smoking history, n (%)	
Current/Former/Never	0 (0)/3 (9.1)/30 (90.9)
Pack-years ≥20, n (%)	1 (3.0)
Alcohol abuse, n (%)	1 (3.0)
Underlying respiratory disease, n (%)	
Old pulmonary tuberculosis	2 (6.1)
Bronchiectasis	2 (6.1)
Interstitial pneumonia	1 (3.0)
Underlying systematic disease, n (%)	
Old cerebral infarction	1 (3.0)
Diabetes mellitus	1 (3.0)
Radiographic features, n (%)	
NB/FC	8 (24.2)/25 (75.8)
Total lesion extent^a^ 1/2/3, n (%)	2 (6.1)/24 (72.7)/7 (21.2)
Cavity at the beginning of treatment, n (%)	25 (75.8)
Laboratory	
White blood cell, /μl ± SD	5590 ± 1437
Neutrophil, /μl ± SD	3915 ± 1291
Hemoglobin, g/dl ± SD	12.4 ± 1.3
Serum albumin, g/dl ± SD	3.8 ± 0.6
CRP, mg/dl ± SD	1.9 ± 2.9
AST, mg/dl ± SD	23 ± 7
ALT, mg/dl ± SD	15.9 ± 8.4
Cre, mg/dl ± SD	0.5 ± 0.14
Chronic infection with *P. aeruginosa*	3 (9.1)
With treatment for pulmonary aspergillosis, n (%)	4 (12.1)
Steroid use, n (%)	0 (0)
Past MAC treatment	
CAM monotherapy, n (%)	6 (18.2)
Two-drug regimen including CAM, n (%)	4 (12.1)
MAC treatment more than 5 years, n (%)	20 (60.6)

Data are presented as no. (%) or mean ± SD

*MAC Mycobacterium avium* complex, *NB* nodular/bronchiectatic disease, *FC* fibrocavitary disease, *CRP* C-reactive protein, *AST* aspartate transaminase, *ALT* alanine transaminase, *Cre* creatinine, *CAM* clarithromycin

^a^1, within one third of unilateral lung field; 2, within unilateral lung field; 3, over unilateral lung field

**Table 2:** MIC breakpoints and in vitro susceptibility of *Mycobacterium avium* complex

	MIC (μg/ml)										
Antibiotics	≦0.125	0.25	0.5	1	2	4	8	16	32	64	≧128
RFP	9	3	9	3	4	3	***1***	***1***			
EB					1		***5***	***12***	***5***	***8***	***2***
LVFX		2	13	6	7	2	***2***	***1***			
KM				2	2	10	***9***	***3***	***1***	***0***	***6***
SM			1	3	9	10	***9***	***1***			
AMK				2	2	9	***9***	***3***	***8***		

*MIC* minimum inhibitory concentration, *RFP* rifampicin, *EB* ethambutol, *LVFX* levofloxacin, *KM* kanamycin, *SM* streptomycin, *AMK* amikacin

Italics and bold type indicate susceptible and resistant categories of interpretive criteria for each antimicrobial agent, respectively

Regimens after isolation of CAM-resistant MAC are summarised in Table 3. Among the 33 enrolled patients, 24 were treated with a CAM-containing regimen, 7 were treated with a regimen without CAM, 2 were treated with an AZM-containing regimen and 21 patients were treated with three or more drugs except macrolides. Of the patients receiving a new quinolone, 16 were treated with LVFX, 2 with STFX and 2 with MFLX. The mean length of aminoglycoside treatment was 5 months. No patients underwent surgical resection.

**Table 3:** Regimens after isolation of CAM-resistant MAC

Regimen		n	Radiological findings: improved or unchanged at one year after treatment	Sputum conversion by one year after treatment
CAM-containing regimen	Total	24	11	8
Plus one drug	RFP	1	1	1
	EB	1	0	0
Plus two drugs	RFP + EB	5	2	3
	RFP + NQ	1	1	0
	EB + NQ	1	0	0
Plus three drugs	RFP + EB + NQ	6	4	1
	RFP + EB + KM	3	1	2
	RFP + KM + NQ	2	1	0
	RFP + NQ + INH	1	0	1
	EB + KM + NQ	1	0	0
Plus four drugs	RFP + EB + KM + NQ	1	0	0
	RFP + EB + KM + INH	1	1	0
AZM-containing regimen	Total	2	1	2
Plus two drugs	RFP + NQ	2	1	2
Regimen without CAM	Total	7	3	2
Monotherapy	EB	1	0	0
Three drugs	RFP + EB + KM	1	0	0
	RFP + EB + NQ	5	3	2

*CAM* clarithromycin, *MAC Mycobacterium avium* complex, *RFP* rifampicin, *EB* ethambutol, *NQ* new quinolone, *KM* kanamycin, *INH* isoniazid, *AZM* azithromycin

The treatment regimen was changed in 28 (85 %) patients after the discovery of CAM-resistant MAC. Antibiotic treatment was discontinued in 4 patients (12 %) between 8 and 10 months after starting the regimen and in 12 patients (36 %) was between 10 and 12 months because of a persistent positive culture despite the administration of antibiotics (*n* = 15), worsening symptoms (*n* = 15), death (*n* = 2) and other causes (*n* = 2). The median duration to be evaluated was 10.4 months (10.4 ± 1.6 months) after beginning the treatment regimen.

### Treatment outcomes and factors associated with treatment response

As shown in Tables 4 and 5, the sputum conversion rate was 36 % (12/33) and the non-deterioration rate was 45 % (15/33). Of the 12 patients with CAM-resistant MAC lung disease whose sputum had converted to negative, 9 could not expectorate sputum at the time of response evaluation. Radiological findings were classified as improved in 4 patients, as unchanging in 11 patients, and as worsening in the remaining patients. One patient, whose sputum cultures had converted to negative and radiological findings had improved, was treated with CAM, RFP, EB and KM. The 1-year mortality of all analysed patients was 6 % (2/33).

**Table 4:** Analysis of the risk factors of radiological non-deterioration evaluated with chest X-ray and CT scans

Variables	Total	Non-deterioration	Worsening	Multivariate analysis
	n	(*n* = 15)	(*n* = 18)	Odds ratio
Continuation of CAM or AZM	26	12 (80)	14 (78)	3.7 (0.28–50)
Treatment with 3 or more drugs	21	10 (67)	11 (66)	1.3 (0.16–11)
Age ≥75 years	8	3 (20)	5 (28)	
Female	31	15 (100)	16 (89)	
Nonsmoker	3	1 (6.7)	2 (11)	
BMI ≧18 kg/m^2^	11	6 (40)	5 (28)	
NB (radiological findings)	27	13 (87)	14 (78)	
Total lesion extent^a^ 1 or 2	26	13 (87)	13 (72)	
Cavity	25	9 (60)	16 (89)	0.22 (0.018–2.7)
Albumin ≥3.5 g/dl	26	14 (93)	12 (67)	6.8 (0.28–168)
CRP <1.0 mg/dl^*^	16	12 (80)	4 (22)	12 (1.6–95)^**^

Data are presented as no. (%)

*CAM* clarithromycin, *AZM* azithromycin, *NB* nodular/bronchiectatic disease, *BMI* body mass index, *CRP* C-reactive protein

^*^Significant at *p* < 0.005 in univariate analysis

^**^Significant at *p* < 0.05 in multivariate analysis

^a^1 within one third of unilateral lung field; 2 within unilateral lung field

**Table 5:** Analysis of the risk factors of microbiological improvement evaluated with MAC cultures

Variables	Total	Sputum conversion	Sputum positive	Multivariate analysis
	n	(*n* = 12)	(*n* = 21)	Odds ratio
Continuation of CAM or AZM	26	10 (83)	16 (76)	1.1 (0.13–8.7)
Treatment with 3 or more drugs	21	6 (50)	15 (71)	0.57 (0.092–3.5)
Age ≥75 years^*^	8	6 (50)	2 (9.5)	5.7 (0.67–49)
Female	31	10 (83)	21 (100)	
Nonsmoker	30	11 (92)	19 (90)	
BMI ≧18 kg/m^2^	11	5 (42)	6 (29)	
NB (radiological findings)	27	9 (75)	18 (86)	
Total lesion extent^a^ 1 or 2	26	9 (75)	17 (81)	
Cavity	25	10 (83)	15 (71)	
Albumin ≥3.5 g/dl	26	11 (92)	15 (71)	3.0 (0.22–41)
CRP <1.0 mg/dl	16	5 (42)	11 (52)	0.79 (0.12–5.2)

Data are presented as no. (%)

*CAM* clarithromycin, *AZM* azithromycin, *NB* nodular/bronchiectatic disease, *BMI* body mass index, *CRP* C-reactive protein

^*^Significant at *p* < 0.05 in univariate analysis

^a^1 within one third of unilateral lung field; 2 within unilateral lung field

Analyses of the factors associated with treatment response are also shown in Tables 4 and 5. First, we performed univariate analyses of the association of different variables. The variables of cavity, serum albumin ≥3.5 mg/dl and C-reactive protein (CRP) <1.0 mg/dl could be associated with radiological non-deterioration. The variables of age ≥75 years and male could be associated with microbiological improvement. In the multivariate analysis, CRP <1.0 mg/dl (*p* = 0.002, odds ratio 12, 95 % confidence interval (CI) 1.6–95) was found to be the only significant risk factor for radiological non-deterioration, and no significant risk factors for microbiological improvement were found. Continuation of macrolides or treatment with three or more drugs except a macrolide was not found to be significant factors for microbiological improvement and radiological non-deterioration.

## Discussion

The main finding of this study was that continuation of macrolides and treatment with three or more drugs except a macrolide seemed not to be factors for radiological and microbiological improvement. The optimal therapeutic regimen has not been established and limited data are available in the literature regarding the clinical effectiveness of regimens to treat CAM-resistant MAC lung disease. To our knowledge, no published studies have focussed on continuation of the macrolide or addition of a new quinolone or injectable aminoglycoside after the emergence of CAM-resistant MAC.

Some experts recommend that the basis of therapy for patients with CAM-resistant MAC lung disease includes EB, RBT, a parenteral agent and, whenever possible, adjunctive surgery [20]. In one large study of 51 patients with macrolide-resistant MAC lung disease, the authors concluded that macrolide-resistant MAC lung disease requires both the use of surgery and administration of parenteral aminoglycosides [6]. However, there are few data on the effectiveness of combination drug therapy for macrolide-resistant MAC lung disease. It is certain that lung resection surgery for mycobacterial disease can be associated with a favourable treatment outcome, but the surgery is potentially associated with significant complications, morbidity and mortality [1, 21, 22]. In addition, although patients whose disease is predominantly localised to one lung might be considered as surgical candidates [1], the affected lesion of many patients had spread from one lung to both lungs after years of treatment with antibiotics for the emergence of CAM-resistant MAC. Therefore, it is important to carefully choose those patients who would best benefit from surgery, and more surveys of the drug treatment of CAM-resistant MAC lung disease are needed.

We could not show significant benefits of the continuation of macrolides in the present study, although there is evidence for the effectiveness of macrolides in the long-term treatment of a spectrum of chronic inflammatory respiratory diseases. The respective non-deterioration rate and the conversion rate were similar in patients with continuation of macrolides compared with those without continuation of macrolides. Although the odds ratio showed continuation to be preferable, the wide 95 % CI might have resulted from the small number of patients in this study. It is thus concluded that there is limited evidence to support continuation of macrolide as treatment for macrolide-resistant MAC lung disease if the patient does not have complications such as one of the chronic inflammatory respiratory diseases. This therapy would be considered worthy of additional study.

Our study also found that combination treatment with three or more drugs except a macrolide was also not a factor for radiological and microbiological improvement. For patients whose isolates become macrolide-resistant, adding drugs, including aminoglycoside and a new quinolone, should be considered because the continuation of the previous drug therapy would not be successful. The addition of aminoglycoside tended to show an increase in the sputum conversion rate, but it did not result in a significant improvement of long-term outcome in macrolide susceptible patients [17]. As for the new quinolones, the evidence of their effect on MAC lung disease has not been fully demonstrated although some studies have reported effectiveness for the treatment of pulmonary MAC disease [23, 24]. These studies suggested that the addition of aminoglycoside or a new quinolone would be beneficial in patients with CAM-resistant MAC lung disease; however, the benefits of combining these drugs against MAC are controversial because of the high incidence of potentially serious adverse effects. Although we could not show their clinical effectiveness in this study, possibly because of the small sample size, further study is required to evaluate the precise clinical effectiveness of these drugs.

No patients were treated with RBT in this study. RBT is more active in vitro than RFP against MAC [25], and RBT is associated with less drug interaction in CAM metabolism and a higher serum concentration than that of RFP [26]. RBT is an effective drug that is clinically similar to RFP [27]. RBT was not used in this study primarily because it causes adverse events including uveitis, leukopenia, nausea and polyarthralgia, which occur infrequently with RFP [28]. Thus, the effectiveness of RBT should be evaluated in further clinical trials.

Previous trials have shown that the risks for the emergence of CAM-resistant MAC are CAM monotherapy and CAM plus companion medications that are inadequate for protection against the emergence of resistance, such as CAM and fluoroquinolone [4, 6]. Only 6 (17.6 %) of our patients underwent monotherapy prior to the emergence of CAM resistance, and many of the susceptible patients were women, had never smoked, had nodular bronchiectatic disease and had received adequate long-term combination therapies. This finding suggests that the isolation of CAM-resistant MAC can occur even if patients are treated with adequate therapies. Therefore, the means to prevent macrolide resistance should be investigated further in patients with long-term treated MAC lung disease.

Our study has several limitations. First, the numbers of radiological examinations and sputum specimens obtained during the before-and-after therapy for CAM-resistant MAC lung disease were relatively low. If these tests were performed more frequently, we would find CAM resistance earlier and also might be able to more accurately evaluate response after therapy. Second, we could not evaluate the patients’ clinical symptoms. Because CAM resistant-MAC lung disease is difficult to cure, it can be reasonable to start treatment to relieve the symptoms; prospective clinical trials would be the proper evaluating approach. Third, the regimen in each patient was based on the decision of the attending physician without an established institutional protocol. However, the average dosage of these regimens could be reasonable for the MAC lung disease patients. Fourth, although previous studies have speculated the importance of the addition of parenteral aminoglycoside [6], the number of patients with aminoglycoside was relatively small. This is because parenteral aminoglycoside had ever been used before the emergence of CAM-resistant MAC or adverse effects of parenteral aminoglycoside had occurred. Fifth, whereas chest CT is superior to chest radiography in evaluating radiological effectiveness, most patients in this study underwent chest radiography before and after treatment for CAM-resistant MAC lung disease. As a result, the true radiological effectiveness might be misjudged. Sixth, the sputum was considered to have converted to negative in this study if the patient could not expectorate sputum. Although this would be generally-accepted definition, the true sputum conversion could be misjudged [5, 17, 19]. Seventh, the timing of judging treatment effectiveness was not fixed (10.4 ± 1.6 months). This was because follow-up interval was based on the decision of the attending physician, usually every two to three months. In addition, some patients discontinued treatment within a year. However, the extent of the difference was considered to be acceptable for the statistical analysis. Finally, the number of cases (*n* = 33) might be too small to detect clinically significant differences between each regimen. Because a large sample size and use of validated methods might have strengthened the data and conclusions, the efficacy of the continuation of macrolides and treatment with three or more drugs except a macrolide should be evaluated in a large clinical trial.

## Conclusions

We could not suggest that continuation of macrolides (CAM, AZM) or addition of a new quinolone or injectable aminoglycoside to combination treatment with RFP and EB improve patients’ clinical outcome after the emergence of CAM-resistant MAC. However, further study is required to definitely evaluate the precise clinical efficacy of these drugs. In addition, because isolation of CAM-resistant MAC can result in poor treatment outcome in spite of drug therapy, prevention of macrolide resistance is highly important as is further research on CAM-resistant MAC lung disease.

## Abbreviations

AMK: amikacin
ATS: American Thoracic Society
AZM: azithromycin
CAM: clarithromycin
COPD: chronic obstructive pulmonary disease
CT: computed tomography
EB: ethambutol
IDSA: Infectious Disease Society of America
KM: kanamycin
LVFX: levofloxacin
MAC: mycobacterium avium complex
MFLX: moxifloxacin
MIC: minimum inhibitory concentration
OR: odds ratio
RBT: rifabutin
RFP: rifampicin
SD: standard deviation
SM: streptomycin
STFX: sitafloxacin

## References

[1] Griffith DE, Aksamit T, Brown-Elliott BA, Catanzaro A, Daley C, Gordin F (2007). An official ATS/IDSA statement: diagnosis, treatment, and prevention of nontuberculous mycobacterial diseases. Am J Respir Crit Care Med.

[2] Prevots DR, Shaw PA, Strickland D, Jackson LA, Raebel MA, Blosky MA (2010). Nontuberculous mycobacterial lung disease prevalence at four integrated health care delivery systems. Am J Respir Crit Care Med.

[3] Winthrop KL, McNelley E, Kendall B, Marshall-Olson A, Morris C, Cassidy M (2010). Pulmonary nontuberculous mycobacterial disease prevalence and clinical features: an emerging public health disease. Am J Respir Crit Care Med.

[4] Wallace RJ, Brown BA, Griffith DE, Girard WM, Murphy DT, Onyi GO (1994). Initial clarithromycin monotherapy for Mycobacterium avium-intracellulare complex lung disease. Am J Respir Crit Care Med.

[5] Tanaka E, Kimoto T, Tsuyuguchi K, Watanabe I, Matsumoto H, Niimi A (1999). Effect of clarithromycin regimen for Mycobacterium avium complex pulmonary disease. Am J Respir Crit Care Med.

[6] Griffith DE, Brown-Elliott BA, Langsjoen B, Zhang Y, Pan X, Girard W (2006). Clinical and molecular analysis of macrolide resistance in Mycobacterium avium complex lung disease. Am J Respir Crit Care Med.

[7] Southern KW, Barker PM, Solis-Moya A, Patel L (2012). Macrolide antibiotics for cystic fibrosis. Cochrane Database Syst Rev.

[8] Kudoh S, Azuma A, Yamamoto M, Izumi T, Ando M (1998). Improvement of survival in patients with diffuse panbronchiolitis treated with low-dose erythromycin. Am J Respir Crit Care Med.

[9] Albert RK, Connett J, Bailey WC, Casaburi R, Cooper JAD, Criner GJ (2011). COPD Clinical Research Network: azithromycin for prevention of exacerbations of COPD. N Engl J Med.

[10] Seemungal TAR, Wilkinson TMA, Hurst JR, Perera WR, Sapsford RJ, Wedzicha JA (2008). Long-term erythromycin therapy is associated with decreased chronic obstructive pulmonary disease exacerbations. Am J Respir Crit Care Med.

[11] Altenburg J, de Graaff CS, Stienstra Y, Sloos JH, van Haren EH, Koppers RJ (2013). Effect of azithromycin maintenance treatment on infectious exacerbations among patients with non–cystic fibrosis bronchiectasis: the BAT randomized controlled trial. JAMA.

[12] Serisier DJ, Martin ML, McGuckin MA, Lourie R, Chen AC, Brain B (2013). Effect of long-term, low-dose erythromycin on pulmonary exacerbations among patients with non-cystic fibrosis bronchiectasis: the BLESS randomized controlled trial. JAMA.

[13] American Thoracic Society and the Centers for Disease Control and Prevention (2000). Diagnostic standards and classification of tuberculosis in adults and children. Am J Respir Crit Care Med.

[14] National Committee for Clinical Laboratory Standards (2011). Susceptibility testing of mycobacteria, Nocardia, and other aerobic actinomycetes.

[15] van Ingen J, Egelund EF, Levin A, Totten SE, Boeree MJ, Mouton JW (2012). The pharmacokinetics and pharmacodynamics of pulmonary Mycobacterium avium complex disease treatment. Am J Respir Crit Care Med.

[16] Kobashi Y, Abe M, Mouri K, Obase Y, Kato S, Oka M (2012). Relationship between clinical efficacy for pulmonary MAC and drug-sensitivity test for isolated MAC in a recent 6-year period. J Infect Chemother.

[17] Kobashi Y, Matsushima T, Oka M (2007). A double-blind randomized study of aminoglycoside infusion with combined therapy for pulmonary Mycobacterium avium complex disease. Respir Med.

[18] The Committee for Classification of Tuberculosis in the Japanese Society for Tuberculosis (1959). The criteria of chest roentgenogram classification of pulmonary tuberculosis established by the Japanese Society for Tuberculosis. Kekkaku.

[19] Jeon K, Kwon OJ, Lee NY, Kim B-J, Kook Y-H, Lee S-H (2009). Antibiotic treatment of Mycobacterium abscessus lung disease: a retrospective analysis of 65 patients. Am J Respir Crit Care Med.

[20] Griffith DE, Aksamit TR (2012). Therapy of refractory nontuberculous mycobacterial lung disease. Curr Opin Infect Dis.

[21] Shiraishi Y, Katsuragi N, Kita H, Hyogotani A, Saito MH, Shimoda K (2013). Adjuvant surgical treatment of nontuberculous mycobacterial lung disease. Ann Thorac Surg.

[22] Mitchell JD, Bishop A, Cafaro A, Weyant MJ, Pomerantz M (2008). Anatomic lung resection for nontuberculous mycobacterial disease. Ann Thorac Surg.

[23] Fujita M, Kajiki A, Tao Y, Miyazaki M, Ouchi H, Harada E (2012). The clinical efficacy and safety of a fluoroquinolone-containing regimen for pulmonary MAC disease. J Infect Chemother.

[24] Koh W-J, Hong G, Kim S-Y, Jeong B-H, Park HY, Jeon K (2013). Treatment of refractory Mycobacterium avium complex lung disease with a moxifloxacin-containing regimen. Antimicrob Agents Chemother.

[25] Kunin CM (1996). Antimicrobial activity of rifabutin. Clin Infect Dis.

[26] Wallace RJ, Brown BA, Griffith DE, Girard W, Tanaka K (1995). Reduced serum levels of clarithromycin in patients treated with multi-drug regimens including rifampin or rifabutin for Mycobacterium avium-M. intracellulare infection. J Infect Dis.

[27] Wallace RJ, Brown BA, Griffith DE, Girard WM, Murphy DT (1996). Clarithromycin regimens for pulmonary Mycobacterium avium complex. The first 50 patients. Am J Respir Crit Care Med.

[28] Shafran SD, Deschênes J, Miller M, Phillips P, Toma E (1994). Uveitis and pseudojaundice during a regimen of clarithromycin, rifabutin, and ethambutol. MAC Study Group of the Canadian HIV Trials Network. N Engl J Med.

